# The many states of STIM1

**DOI:** 10.7554/eLife.75174

**Published:** 2021-12-07

**Authors:** Marc Fahrner, Christoph Romanin

**Affiliations:** 1 Institute of Biophysics, Johannes Kepler University Linz Linz Austria

**Keywords:** calcium signaling, store-operated calcium entry, STIM1, smFRET, cell signaling, Human

## Abstract

Harnessing single-molecule FRET illuminates the structural changes necessary for a protein to fine-tune the influx of calcium when reserves inside a cell run low.

**Related research article** van Dorp S, Qiu R, Choi UB, Wu MM, Yen M, Kirmiz M, Brunger AT, Lewis RS. 2021. Conformational dynamics of auto-inhibition in the ER calcium sensor STIM1. *eLife* **10**:e66194. doi: 10.7554/eLife.66194

Over the past decades, the humble calcium ion has emerged as a crucial intracellular messenger that participates in an impressively diverse array of biological processes, from fertilization to programmed cell death. It plays a key role in cells across the body, helping to activate the immune system, release neurotransmitters, and trigger muscle contractions. Imbalances in the concentration of calcium inside a cell leads to chaotic responses and, in some cases, to diseases that can sometimes be fatal (Feske et al., 2006; Lacruz and Feske, 2015; Morin et al., 2020).

The flow of calcium ions into cells is therefore tightly regulated, including by a system known as SOCE (short for store operated calcium entry), which has recently been gaining attention (Prakriya and Lewis, 2015). When calcium levels drop inside an intracellular storage compartment called the endoplasmic reticulum, SOCE increases the intake of calcium ions across the plasma membrane that separates the cell from its environment. The system relies on two proteins: Orai, a calcium-selective channel studded through the plasma membrane; and the stromal interaction molecule (STIM1), located in the membrane surrounding the endoplasmic reticulum (Prakriya and Lewis, 2015). STIM1 can sense the levels of calcium inside the reticulum and activate Orai when these get low. For precisely the right amount of calcium to be ushered into the cell, STIM1 and Orai must be tightly regulated. Now, in eLife, Richard Lewis and colleagues based at Stanford University School of Medicine – including Stijn van Dorp as first author – report impressive insights into the conformations and dynamics of STIM1 (van Dorp et al., 2021).

In its resting state, STIM1 forms a dimer that binds to calcium ions present in high concentrations inside the reticulum and adopts a tightly packed conformation (Figure 1A, left). One tail of the STIM1 protein features three large ‘coiled-coil’ domains (CC1, 2 and 3), which are stabilized in the resting, tight state via a specific type of ‘coiled-coil’ interaction (Fahrner et al., 2020). Extracellular signals such as growth factors, hormones or foreign molecules can trigger calcium-dependent signaling pathways and therefore deplete reticulum calcium reserves. As a result (Zhou et al., 2013), STIM1 loses the ions it is bound to, and undergoes a series of conformational changes that culminate in its cytoplasmic portion (which is in contact with the inside of the cell) being extended, and increasing in length (Figure 1A, right). This elongated STIM1 stretches to the edge of the cell, reaching junctions that bring together the plasma membrane and the reticulum. Once there, it encounters and couples to Orai, triggering the channel to open and allow calcium ions to enter the cell (Prakriya and Lewis, 2015).

**Figure 1.**
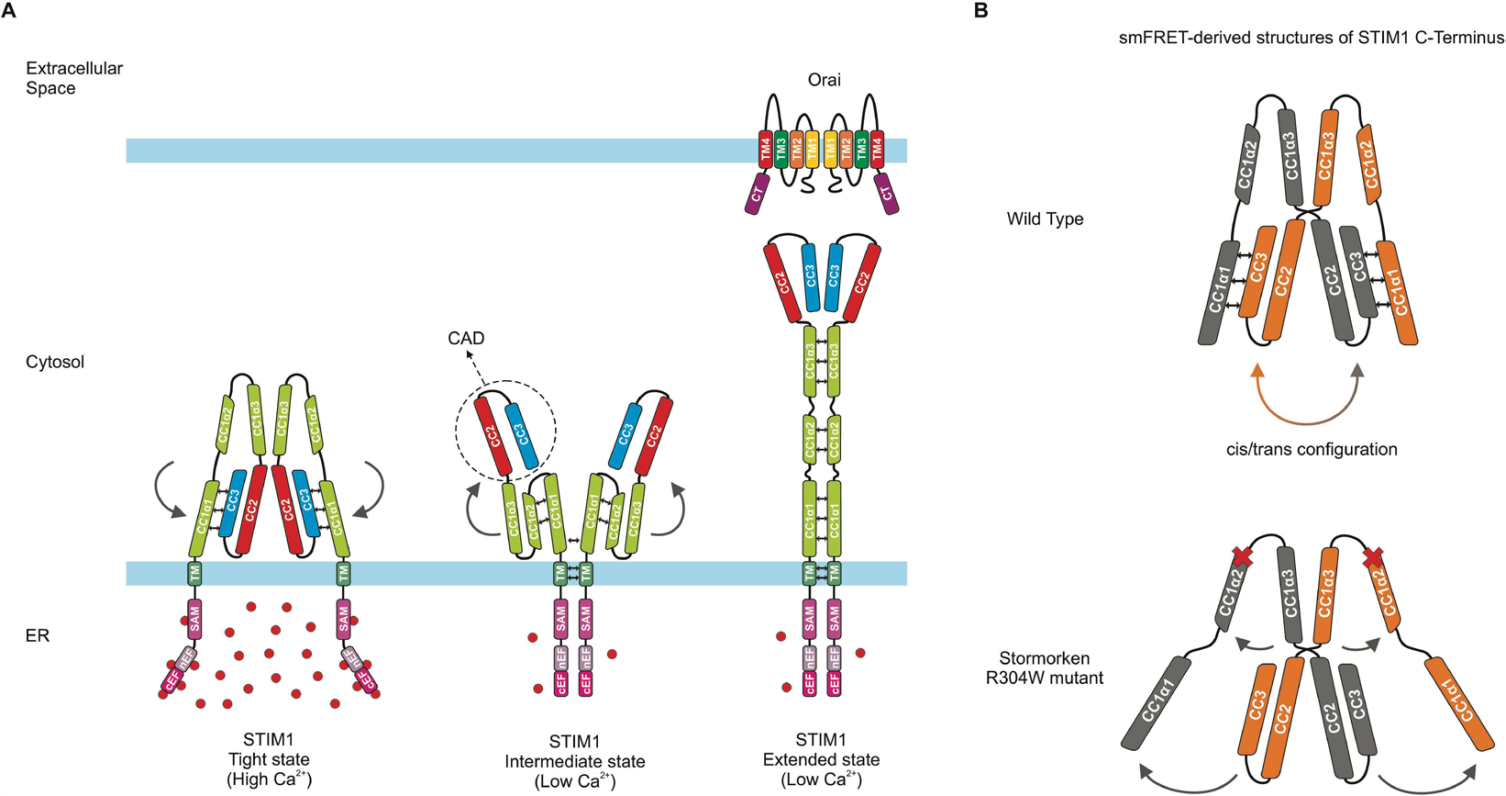
Conformation and dynamics of STIM1. (**A**) Models of dimeric STIM1 conformational states. (Left) When the endoplasmic reticulum (ER) calcium store is full, a subdomain of CC1 (called α1) and the CC2-CC3 domains of CAD form a tight coiled-coil interaction (represented by small black double arrows), which is characteristic of the tight, resting state of STIM1 and prevents binding to Orai. (Middle) Upon calcium store depletion, the STIM1 domains inside the ER (pink) lose their bound Ca^2+^ ions (red spheres), causing the regions to interact with one another and eliciting an interaction (double arrows) between the transmembrane domains (TM; dark green) of the dimerized STIM proteins. These rearrangements likely lead to an intermediate state in which the CC1α1-CAD coiled-coil interaction is released (represented by curved arrows), and CAD is projected towards the plasma membrane while CC1α1 comes into close proximity to the other two subdomains of CC1 (α2 and α3). (Right) CC1 then fully unfolds into its extended state, allowing CAD to interact with and activate Orai. (**B**) (Top) Schematic showing the dimeric STIM1 C-terminus structure derived by van Dorp et al. from smFRET data. Double arrows between the domains (orange and grey) indicate switching of CC1α1-CAD interaction between intramolecular (’cis‘) and intermolecular (’trans’) within the STIM1 C-terminus dimer. (Bottom) The mutation associated with Stormorken syndrome disrupts the connection between CC1α1 and CAD, as revealed by the smFRET data. Dark grey arrows show how the position of the STIM1 domains changes, and red crosses mark the position of the mutation (called R304W) at the end of the CC1α2 domain. The STIM1 C-terminal regions downstream of CAD were omitted for simplicity.

Image credit: Herwig Grabmayr (CC BY 4.0).

The CC2 and CC3 domains of STIM1, called CAD (or SOAR), form the Orai-activating region, the structure of which has been examined in dimeric form using X-ray crystallography (Park et al., 2009; Yang et al., 2012; Yuan et al., 2009); yet many aspects remain unknown about how STIM1 can switch between a resting and activated state, how it can activate Orai, and in particular how CC1 interacts with CAD to regulate the activity of this calcium sensor. To shed light on these dynamics, the team harnessed single-molecule FRET microscopy (smFRET), a biophysical technique that allows scientists to measure distances between amino acids using fluorescent compounds; this approach was applied to the C-terminus of STIM1. The resulting data fit well with the previously reported CAD crystal structure, while adding details on the orientation of the domain, in particular its Orai binding region (Baraniak et al., 2020).

Consistent with a recent report, the data suggests that CAD sequesters the Orai binding region near the reticulum membrane (Figure 1A, left; 1B, top; Höglinger et al., 2021). The experiments showed that the coiled-coil domains CC1 and CC3, located on the cytoplasmic side of the reticulum membrane, interact to create an inhibitory clamp that stabilizes STIM1 in its compact, resting state (Figure 1A, left). Previously, an intramolecular, ‘cis’ CC1/ CC3 interaction had been assumed (Fahrner et al., 2014; Ma et al., 2015), but the smFRET data suggested both a switching intermolecular, ‘trans’ as well as intramolecular, ‘cis’ interaction within a STIM1 dimer (Figure 1B, top).

Furthermore, van Dorp et al. focused on the CC1 domain and its three CC1α1, α2 and α3 subregions, confirming the findings of previous studies (Fahrner et al., 2014). Another group used nuclear magnetic resonance spectroscopy to examine both the whole CC1 domain, and how its subregions interact to affect STIM1 activation (Rathner et al., 2021). The structure identified in this study, however, was not observed by van Dorp et al., who suggest it is an intermediate state following STIM1 activation (Figure 1A, middle). Experiments artificially linking together nearby amino acids gave additional structural information about the CC1 subdomains, showing that activated STIM1 appears to shift to a fully elongated conformation to couple to Orai (Figure 1A, right).

Further insights into STIM1 structures were then provided in a disease context, as van Dorp et al. imaged a mutated version of STIM1 associated with a rare condition known as Stormorken syndrome (Morin et al., 2020). The smFRET approach revealed that this change had substantial impact on the conformation of STIM1 (Figure 1B, bottom). In particular, it destabilized the resting, tight state of STIM1 C-terminus by releasing the inhibitory clamp between CC1 and CC3 (Figure 1B).

Without high-resolution structural information, smFRET is an excellent method to grasp the configuration and dynamic of proteins – as long as, of course, the fluorescent molecules required for the technique do not disturb these interactions. Applying this method to a whole STIM1 protein reconstituted into a lipid vesicle would further improve the approach, allowing researchers to assess STIM1 conformational changes under more physiological conditions. How the protein rearranges itself when calcium levels drop in the reticulum could be visualized on both sides of the membrane of this compartment. Orai domains (or even the full channel) could be reconstituted in other vesicles, and then used to probe the interaction between STIM1 and Orai. A finer understanding of the conformational changes of STIM1 as it couples to Orai will shed light on how calcium levels are regulated in both healthy and diseased cells.
